# Acute kaempferol ingestion lowers oxygen uptake during submaximal exercise and improves high‐intensity exercise capacity in well‐trained male athletes

**DOI:** 10.14814/phy2.70369

**Published:** 2025-05-09

**Authors:** Koichi Okita, Tsubasa Mizokami, Osamu Yasuda, Yasutaka Ikeda

**Affiliations:** ^1^ Hokusho University Graduate School of Lifelong Sport Bunkyodai Ebetsu Hokkaido Japan; ^2^ Saga Nutraceuticals Research Institute, Nutraceuticals Division Otsuka Pharmaceutical Co., Ltd. Yoshinogari‐cho, Saga Japan; ^3^ Department of Sports and Life Sciences National Institute of Fitness and Sports in Kanoya Kanoya, Kagoshima Japan; ^4^ Advanced Research Institute for Core Science, Nutraceuticals Division, Otsuka Pharmaceutical Co., Ltd. Konan, Minato‐ku, Tokyo Japan

**Keywords:** ergogenic aid, highland, hypoxia, kaempferol, oxidative metabolism, phytochemical

## Abstract

Our previous study involving 314 highland crop species demonstrated that kaempferol, a flavonoid present in higher amounts in these species, significantly enhances mitochondrial metabolism and cellular ATP production in myoblasts under limited oxygen conditions. Notably, biologically active substances in these plants, such as phytochemicals, may help improve the health and physical strength of highland residents. Therefore, we hypothesized that kaempferol would affect oxygen availability during exercise and exercise performance in vivo. This randomized, double‐blind, placebo‐controlled crossover study aimed to assess the effect of a single kaempferol intake (10 mg) on cardiopulmonary response during submaximal exercises (25%, 50%, and 75% VO_2max_) and maximal and super‐maximal endurance capacities (100% and 125% VO_2max_) in 16 well‐trained male university athletes using constant‐load exercise tests (VO_2max_ 57.5 ± 5.4 mL·kg^−1^ min^−1^). Kaempferol significantly reduced the VO_2_ and respiratory rate during 25%, 50%, and 75% VO_2max_ exercises (*p* < 0.05 vs. placebo) without elevating the respiratory quotient and blood lactate. It also significantly increased the exercise duration at 100% VO_2max_ (*p* < 0.05 vs. placebo). Overall, we demonstrated for the first time that a single intake of kaempferol could reduce oxygen uptake (demand/cost) during constant‐load submaximal exercise and extend time‐to‐exhaustion during maximal exercise. UMIN Clinical Trials Registry in Japan: UMIN000049589.

## 1 INTRODUCTION

Plants biosynthesize valuable phytochemicals in response to environmental stress, including food components such as flavonoids and carotenoids, which eliminate or remove free radicals formed during UV exposure (Dinkova‐Kostova, 2008). Furthermore, plants produce phytochemicals with higher biological activity under hypoxic conditions than under normal oxygen levels (Mizokami et al., 2021). Therefore, the specific compounds in these plants may contribute to enhancing the physical health of high‐altitude residents. We previously conducted a screening test for the cellular ATP production enhancement abilities of 65 phytochemicals from 314 crop extracts from plants mainly cultivated at high altitudes in a low‐oxygen cell evaluation system that mimics the biological environment. Notably, kaempferol, which is abundant in high‐altitude foods (cabbage, >100 times greater than low‐altitude equivalent; Japanese white radish, >10 times greater; and potherb mustard, ~2 times greater), significantly increases intracellular ATP levels by activating mitochondrial metabolism (Akiyama et al., 2022; Mizokami et al., 2021). Kaempferol is a natural flavonoid found in various vegetables, fruits, and beverages (Calderon‐Montano et al., 2011; Mizokami et al., 2021) that exhibits strong antioxidant properties (Singh et al., 2008; Tzeng et al., 2011). We speculated that kaempferol might support the physical abilities and health of high‐altitude residents. Thus, in our previous study, we demonstrated that a single oral dose of kaempferol reduces cardiopulmonary load during 400 m runs (Okita et al., 2024). Consequently, kaempferol was hypothesized to reduce oxygen demand/cost during exercise, alleviate cardiopulmonary strain, and improve exercise capacity by enhancing ATP production efficiency in vivo, similar to that observed in vitro. However, studies on the effects of kaempferol intake on oxygen uptake, cardiopulmonary responses during exercise, and exercise performance are lacking.

Therefore, this study aimed to investigate for the first time the effect of a single oral dose of kaempferol (10 mg) on the cardiopulmonary burden, including ventilatory gas and blood analysis during submaximal fixed‐load exercise and exercise capacity during supramaximal exercises in well‐trained athletes using a bicycle ergometer.

## 2 MATERIALS AND METHODS

### 2.1 Ethical declarations

The study protocol complied with the guidelines of the Declaration of Helsinki and was approved by the Research Ethics Committee of the National Institute of Fitness and Sports in Kanoya (approval no. 15–85) and the Ethics Review Board of the Otsuka Pharmaceutical Research Institute (approval no. 1805). This study was also registered with the UMIN Clinical Trials Registry of Japan (registry no. UMIN000049589). Written informed consent was obtained from all participants after the experimental procedures and their associated risks were explained.

### 2.2 Test supplement ingestion and blinding

To confirm that the two capsule types used in this study were indistinguishable, the relevant identification codes were printed on a pack containing each substance, sealed in an envelope, and delivered to an allocator, who further confirmed that the capsule could not be identified based on appearance or smell. The allocator then replaced the kaempferol supplementation and placebo codes with control codes that could not be easily identified, thereby blinding the study. The products were then transferred to the test product manager. The control codes were sealed in an envelope with the corresponding table and retained with the allocator until the study was unblinded. The test supplement was administered as a capsule, and kaempferol was substituted with cornstarch as a placebo. Kaempferol aglycone used in this study was prepared by converting the kaempferol glycoside of quinoa powder using aromatase treatment. The mass and amounts of nutrients and calories were similar in both the active and placebo capsules. On each testing day, the participants ingested an active capsule (containing 10 mg kaempferol) or a placebo capsule (containing <0.001 mg kaempferol) in a randomly assigned order in the morning in a fasting state. Each capsule was ingested in a single dose, and the subsequent test capsule was ingested after ≥3 days to ensure sufficient wash‐out confirmed via preliminary laboratory examination. Capsules used in this study were provided by Otsuka Pharmaceutical Co., Ltd. (Tokyo, Japan). The participants and researchers were blinded after being assigned to the interventions by an independent test food allocation manager. In preliminary studies, the enhancement of exercise capacity following kaempferol intake plateaued between 10 mg and 50 mg. Consequently, a 10 mg dose was selected for the present study. Notably, several clinical trials have demonstrated the safety of kaempferol in humans (Akiyama et al., 2023; Ikeda et al., 2024; Ikeda et al., 2024; Okita et al., 2024).

### 2.3 Participants, eligibility criteria, and randomization

**FIGURE 1:**
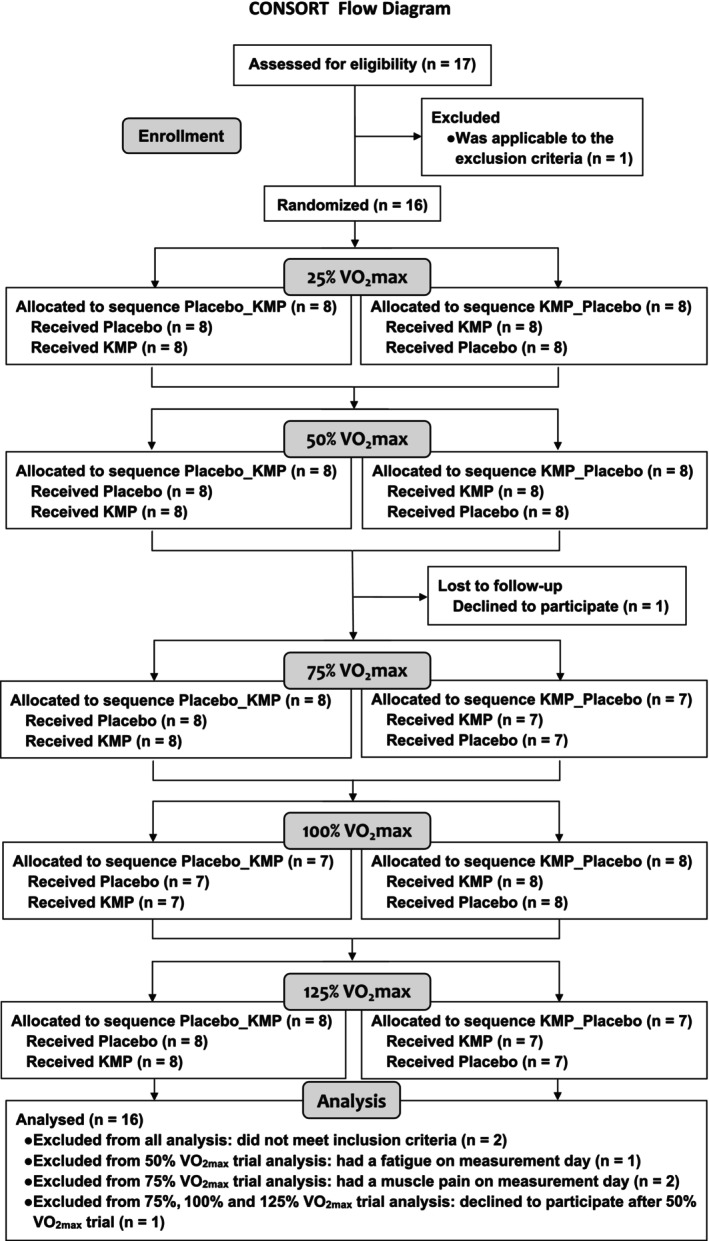
CONSORT flow diagram of the crossover design of this randomized, double‐blind, placebo‐controlled study.

Healthy male university athletes were recruited for this study. All participants visited the laboratory at least 1 week prior to the clinical trial, and a physician conducted their medical examinations, including blood and urine tests. Participant eligibility was determined based on the following inclusion criteria: healthy and well trained on a bicycle ergometer for at least 1 h on at least 5 separate occasions per week. During the follow‐up visit, the participants performed a pre‐exercise test to determine their VO_2max_ (maximal oxygen uptake) capacities. Finally, 17 eligible participants were enrolled in this double‐blind, placebo‐controlled, crossover study for each exercise intensity, and 16 were included in the analysis (Figure 1). The permuted block method (block size, four) was employed for randomization by entering the described variables (age, weight, and VO_2max_).

### 2.4 Experimental design

**FIGURE 2:**
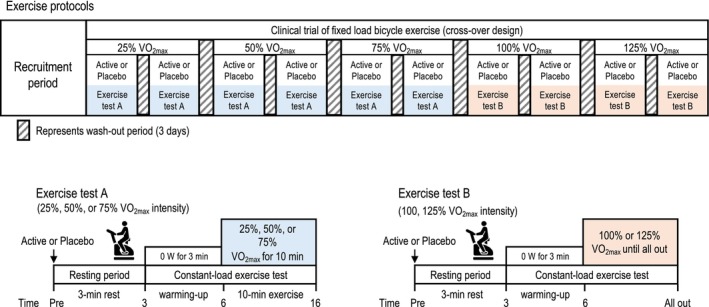
Schematic of the crossover design of a randomized, double‐blind, placebo‐controlled study. Active represents a kaempferol capsule (10 mg). The study involved five different constant‐load exercise tests (upper). Submaximal exercises were performed to measure cardiopulmonary response (lower left), whereas maximal and super‐maximal exercises were performed to measure endurance capacity (lower right). Each exercise protocol was performed after 3 h of kaempferol or placebo supplementation.

The participants visited the laboratory in the morning in a fasted state, consumed supplements (placebo‐ or kaempferol‐containing capsules), and underwent constant‐load bicycle exercise tests after 3 h. In a previous study, we confirmed that the plasma concentration of kaempferol peaks 3 h after ingestion and persists for up to 8 h (Ikeda et al., 2024; Okita et al., 2024), with the washout period set to 3 days. The protocol was repeated at five exercise intensities: 25%, 50%, 75%, 100%, and 125% VO_2max_ (Figure 2). The ratio of kaempferol concentration in the plasma 24 h after the intake/peak time (3 h) was less than 1%. Therefore, 3 days is sufficient for washout after a single‐dose intake and does not appear to result in tissue‐level adaptations, which occur after chronic intake.

Exercise intensities were determined based on the VO_2max_ measured using the Åstrand test (ÅStrand & ÅStrand, 2003; Poole & Jones, 2017) with a bicycle ergometer (Model 874E, Monark Exercise AB, Vansbro, Sweden) during the preliminary test. Pre‐tests, including VO_2_ during an 8 min cycling exercise at a constant intensity at five or more different VO_2max_ intensities, were conducted to determine the relationship between steady‐state oxygen uptake (VO_2_) and exercise intensity for each participant. The pedaling frequency was maintained at 70 rpm, and participants were allowed to rest for approximately 10 min between exercise bouts. The participants who did not completely recover to perform the next high‐intensity exercise were allowed to rest. Following the establishment of a linear relationship between exercise intensity and steady‐state VO_2_ for each participant, VO_2_ was measured during several bouts of exercise at higher intensities to ensure the leveling‐off of VO_2_. As some participants could not maintain the exercise intensity for the entire 8 min, VO_2_ was measured every 30 s from the 3rd to 5th min of exercise until the end, and the highest VO_2_ value was adopted as the VO_2_ at that intensity in such cases. VO_2max_ was determined using the leveling‐off criterion, and leveling‐off of VO_2_ was observed in all participants. Exercise intensities corresponding to 25%, 50%, 75%, 100%, and 125% of VO_2max_ were estimated individually by interpolating the linear relationship between VO_2_ and exercise intensity. Three tests were fixed‐volume exercises of submaximal intensity, and two were supramaximal exercises until exhaustion. The participants completed 10 min of constant‐load bicycle exercises at 60 rpm at 25%, 50%, and 75% VO_2max_ intensities, whereas the maximal and super‐maximal exercises were performed at 100 rpm at 100% and 125% VO_2max_. The program was terminated when the number of rotations decreased to below 95 rpm. Pulmonary gas exchange and ventilation were measured using the Douglas bag method during all the exercise tests. Gas analysis was performed using a mass spectrometer (ARCO‐2000, ARCO System Inc., Chiba, Japan) for respiratory analysis and bioprocess monitoring. Oxygen uptake, minute ventilation (VE), and respiratory quotient (RQ) were measured. The respiratory rate per minute was obtained by video recording the events and counting the movement of the flow sensor. Heart rate was measured using a DYNASCOPE (DS8600 system, FUKUDA DENSHI, Tokyo, Japan). The rate of perceived exertion (RPE) was evaluated using the Borg scale and visual analog scale (VAS) at the end of each exercise. Blood was collected from the antecubital fossa vein before and after the test using a disposable blood collection needle, an adapter, and a vacuum blood collection tube. The collected blood was used to measure the plasma kaempferol and lactate concentrations. Urine samples were stored for 24 h after ingestion of the test supplement, and the absorbed kaempferol content was measured.

Blood lactate levels were measured using the enzymatic method, followed by rapid centrifugation at 3000 rpm for 5 min. Kaempferol concentrations in plasma and urine were measured at Otsuka Pharmaceutical Co., Ltd. The samples were deconjugated using β‐glucuronidase and extracted using solid‐phase extraction. The extracted samples were quantitatively analyzed using liquid chromatography–mass spectrometry (Nexera X2, Shimadzu, Kyoto, Japan; API 3000, Applied Biosystems, CA, USA). The total urinary kaempferol concentration and urine volume were measured and calculated using the following formula:

$$\begin{array}{c} \text{Urinary}\;\text{kaempferol}\;\text{excretion}\;\left(\mathrm{mg}\right)\\ \;=\text{total}\;\text{urinary}\;\text{kaempferol}\;\text{concentration}\;\left(\mathrm{\mu M}\right)\\ \;\times \text{urinary}\;\text{volume}\;\left(L\right)\times 286.24\;(\text{molecular}\;\text{weight}\;\\ \;\text{of}\;\text{kaempferol})\times 1/1000 \end{array}$$

### 2.5 Statistical analysis

Variables were expressed as the mean ± standard deviation (SD) or standard error (SE). Statistical analyses were performed using SAS software (version 9.4, SAS Institute, Cary, NC, USA), and *p* < 0.05 was considered statistically significant. A linear mixed‐effects model with fixed terms fitted for treatment, period, and sequence and a random‐effects model for participant‐within‐sequence were employed to assess changes due to different treatments. Notably, missing data were not included in the primary model because we used a mixed model of repeated measures. Statistical analyses were performed for each exercise intensity level. We calculated the necessary sample size using SAS software, targeting a power of 0.80 to detect a mean difference of 1.4 mL·kg^₋1^ min^−1^ in 25% VO_2max_ with a standard deviation of 1.1 mL·kg⁻^1^ min^−1^, based on prior findings (Ikeda, Gotoh‐Katoh, Yamamoto, et al., 2024). The minimum necessary sample size to observe the impacts was 12.

## 3 RESULTS

### 3.1 Participants

Table 1 summarizes the characteristics of the participants. Plasma and urinary kaempferol concentrations were not detected before kaempferol capsule intake. The VO_2max_ of all participants was >50 mL·kg^−1^ min^−1^.

**TABLE 1:** Participant characteristics.

Parameters	
*N*	16
Age (years)	21.9 ± 0.8
Height (cm)	174.3 ± 6.7
Weight (kg)	68.8 ± 7.0
Body mass index (kg·m^−2^)	22.6 ± 0.9
VO_2_max (mL·kg^−1^ min^−1^)	57.5 ± 5.4
Maximum plasma KMP concentration (μM)	0.238 ± 0.070
Urinary kaempferol excretion (mg)	0.458 ± 0.212

*Note*: Data are presented as the mean ± SD.

### 3.2 Pulmonary ventilation and gas exchange

**FIGURE 3:**
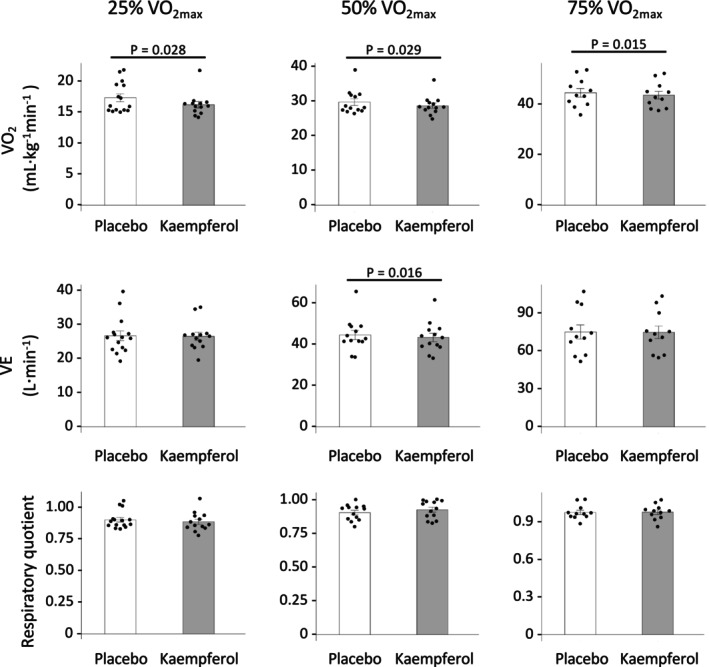
Results of the pulmonary ventilation and gas exchange for five submaximal constant‐load exercises. Data are presented as the mean ± SE.

Figure 3 shows the pulmonary ventilation and gas exchange of VO_2_ at the 25%, 50%, and 75% VO_2max_ exercise intensities. Kaempferol intake at each exercise significantly reduced VO_2_ (25% VO_2max_, 1.4 mL·kg^−1^ min^−1^; 50% VO_2max_, 1.0 mL·kg^−1^ min^−1^; 75% VO_2max_, 0.9 mL·kg^−1^ min^−1^) compared with that using a placebo. VE significantly reduced at 50% VO_2max_ following kaempferol intake. No significant difference was observed in the RQ between kaempferol and placebo intake.

### 3.3 Cardiopulmonary response and blood lactate

**FIGURE 4:**
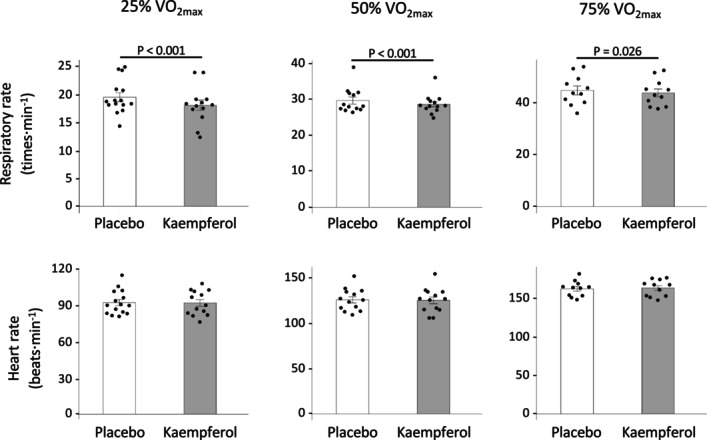
Cardiopulmonary response during five submaximal constant‐load exercises. Data are presented as the mean ± SE.

**FIGURE 5:**
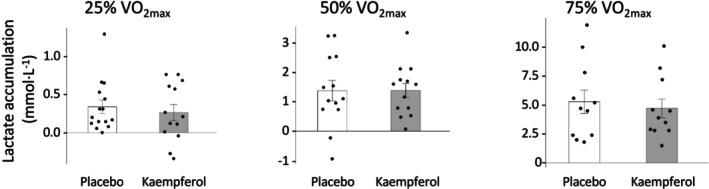
Results of blood lactate levels during constant‐load exercises. Data are presented as the mean ± SE.

Figure 4 shows the cardiopulmonary responses at each exercise intensity level. Kaempferol intake significantly reduced the respiratory rate (25% VO_2max_, 1.9 times·min^−1^; 50% VO_2max_, 1.8 times·min^−1^; 75% VO_2max_, 2.0 times·min^−1^) compared with the placebo. However, significant differences in heart rate were not observed. Furthermore, the blood lactate levels did not differ significantly between the kaempferol and placebo intake groups (Figure 5, Table S1).

### 3.4 Supramaximal exercises

**FIGURE 6:**
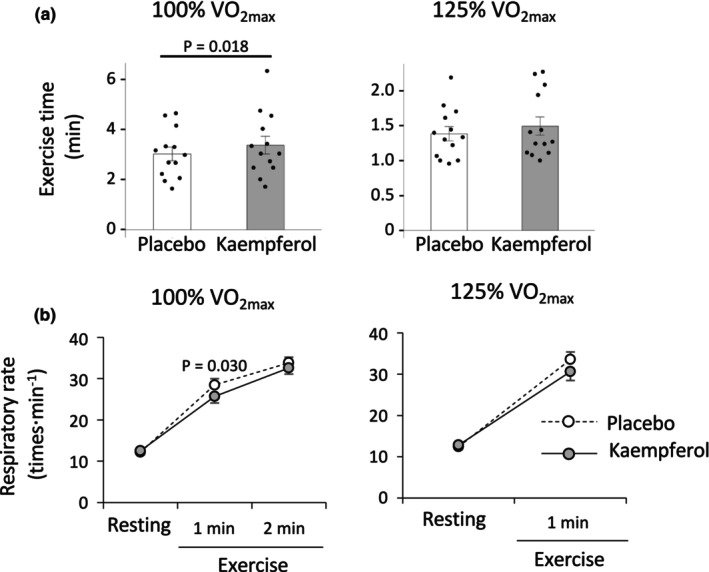
Results of exercise time (a) and respiratory rate (b) during the supramaximal constant‐load exercises. Data are presented as the mean ± SE.

Figure 6 shows the results of supramaximal exercises at 100% and 125% VO_2max_. Kaempferol intake significantly increased exercise duration by 20 s compared with the placebo at 100% VO_2max_ exercise intensity but exhibited no significant change at 125% VO_2max_ exercise intensity (Figure 6a). Kaempferol intake also reduced the respiratory rate at 1 min during 100% VO_2max_ exercise (Figure 6b). No differences were observed in the RPE and VAS scores for muscle fatigue, whereas the VAS score for shortness of breath tended to decrease following kaempferol intake compared with the placebo at 25% and 50% VO_2max_ (Table 2).

**TABLE 2:** Perceived exhaustion during exercises.

Variables	Conditions	Numerical values		
		25% VO_2max_	50% VO_2max_	75% VO_2max_
VAS (cm)				
Muscle fatigue	Placebo	1.1 ± 0.2	3.9 ± 0.6	5.9 ± 0.7
	Kaempferol	0.9 ± 0.3	3.4 ± 0.7	6.4 ± 0.8
Shortness of breath	Placebo	1.3 ± 0.4	3.0 ± 0.3	6.6 ± 0.6
	Kaempferol	0.5 ± 0.1	1.7 ± 0.4	4.7 ± 0.7
RPE				
Tightness of the entire exercise	Placebo	7.8 ± 0.5	11.7 ± 0.6	15.1 ± 0.6
	Kaempferol	8.0 ± 0.5	11.6 ± 0.6	15.4 ± 0.5

*Note*: Data are presented as the mean ± SE.

Abbreviations: RPE, rate of perceived exertion using the Borg scale; VAS, visual analog scale.

## 4 DISCUSSION

We demonstrated for the first time that kaempferol significantly decreased oxygen uptake and respiratory rate during mild‐, moderate‐, and high‐intensity fixed‐load submaximal exercises in well‐trained male athletes, indicating that kaempferol reduces oxygen cost and/or may affect muscular mechanical efficiency during exercise in vivo. Moreover, the lack of differences in the RQ and lactate accumulation further supports this finding, indicating no overactivation of anaerobic metabolism. Kaempferol intake also significantly increases exercise tolerance at 100% VO_2max_, possibly due to an improved submaximal exercise economy (Coyle, 1995). Notably, these results are the first of a series of valuable findings revealed by dietary nitrate intake (Bailey et al., 2009; Jones, 2014; Larsen et al., 2007) and indicate that kaempferol may be a beneficial supplement for athletes. Kaempferol intake could potentially improve athletic ability by reducing the cardiopulmonary load during sports activities.

Kaempferol is a natural flavonoid found in various vegetables, fruits, and beverages (Calderon‐Montano et al., 2011; Mizokami et al., 2021) that exhibits antioxidant properties (Singh et al., 2008; Tzeng et al., 2011). Polyphenols such as flavonoids act as potent antioxidants (Dinkova‐Kostova, 2008; Luo et al., 2019) and activate the nitric oxide (NO) signaling pathway (d'Unienville et al., 2021; Serreli & Deiana, 2023). Experiments have shown that polyphenols, including kaempferol, enhance NO production by increasing endothelial NO synthase (eNOS) expression/activity, inhibiting inducible NO synthase (iNOS) expression, and promoting NO bioavailability via their antioxidant effects, thereby protecting NO from breakdown by ROS (García‐Mediavilla et al., 2007; Hu et al., 2020; Rostoka et al., 2010; Stoclet et al., 2004; Xiao et al., 2009). NO is a ubiquitous molecule involved in numerous physiological processes that improve exercise performance (Jones et al., 2021; Pawlak‐Chaouch et al., 2016). Dietary intake of nitrate, a donor of NO, reduces oxygen demand/cost during exercise (Bailey et al., 2010; Jones, 2014; Larsen et al., 2011). Improvements in mitochondrial efficiency due to dietary nitrate intake, which has been suggested to reduce proton leaks/slippage and improve thermodynamic‐energetic coupling, have been demonstrated (Bailey et al., 2010; Jones, 2014; Larsen et al., 2011). Thus, it would be unsurprising if the flavonoid kaempferol, as a polyphenol, promotes NO bioavailability and might have the same effect as the NO donor of dietary nitrate.

Improvement in mechanical efficiency is presumed to be another important mechanism. Reducing the ATP cost could also reduce the cardiopulmonary load (Larsen et al., 2007). The ATP cost of skeletal muscle contraction is the total ATP consumed by the interaction between myocytes (actomyosin‐ATPase) and Ca^2+^ pumping into the sarcoplasmic reticulum (Ca^2+^‐ATPase) (Barclay et al., 2007). The flavonoid quercetin, a homologue of kaempferol, suppresses Ca^2+^‐ATPase activity and enhances Ca^2+^ release channel activity (Kim et al., 1983; Lee et al., 2002). The increased sensitivity of myocytes to Ca^2+^ can positively affect the mechanical efficiency and strength of skeletal muscles (Bazzucchi et al., 2019; Kim et al., 1983; Lee et al., 2002). Mechanical efficiency improvements have also been reported for the NO‐mediated mechanism (Bailey et al., 2010; Jones, 2014).

Moreover, the flavonoid quercetin may block adenosine receptors at the central level, which may also be involved in the energetics of neurotransmitter uptake and could influence motor unit recruitment capacity, thereby contributing to mechanical efficiency and strength (Davis et al., 2009). Accordingly, its homologue kaempferol may have similar functions. Similar to caffeine, flavonoids affect the central nervous system. Moreover, their effects may be linked to positive emotions, toughness, and upsurge and can affect physical performance (Alexander, 2006; Davis et al., 2003).

Kaempferol and certain polyphenols can enhance mitochondrial biogenesis via the peroxisome proliferator‐activated receptor γ coactivator 1α and nuclear respiratory factor 2/Kelch‐like ECH‐associated protein 1 pathway (Alshehri et al., 2022; Beekmann et al., 2015; Hussain et al., 2022; Wood Dos Santos et al., 2018). Moreover, mitochondrial biogenesis might potentially improve mitochondrial efficiency (Bailey et al., 2010; Holloszy & Coyle, 1984; Jones, 2014). However, the present results were obtained with a single dose of kaempferol; thus, they were not likely associated with enhanced mitochondrial biogenesis.

Considering the results of our study for kaempferol and those of previous studies for dietary nitrate, ATP and oxygen cost reductions may occur simultaneously with a reduction in anaerobic metabolism. This reduction in energy load during exercise by improving mitochondrial and/or mechanical efficiency may not activate lactate production or accumulation. Our study and previous studies using dietary nitrate demonstrated a reduction in energy cost without moderating anaerobic metabolism (Bailey et al., 2009; Larsen et al., 2007). One possible mechanism for oxygen cost reduction may also involve effects outside the mitochondria. Experiments using C_2_C_12_ myotubes suggested that kaempferol stimulates glycolysis (Mizokami et al., 2021). Moreover, polyphenols, including kaempferol, enhance glucose uptake and GLUT4 translocation in experimental muscle cells (Kitakaze et al., 2020; Williamson & Sheedy, 2020; Zygmunt et al., 2010). Therefore, kaempferol may stimulate glycolysis disproportionately with respect to the energy load. Although the amount of ATP provided by glycolysis is small, it may contribute to the reduction of oxygen cost. Taken together, the reductions in ATP and oxygen costs do not necessarily lead to a reduction in lactic acid production. NO has also been shown to increase skeletal muscle glucose uptake during exercise (Hong et al., 2014).

A noticeable decrease in respiratory rate was observed when the cardiopulmonary load was reduced, suggesting that kaempferol may affect airway and respiratory functions during exercise. Polyphenols may also enhance respiratory functions in terms of forced vital capacity and forced expiratory volume (Pounis et al., 2018). Additionally, the strong antioxidant activity and various anti‐allergic effects of kaempferol may be effective against inflammatory disorders, including bronchial asthma, and may stabilize airway hypersensitivity during exercise (Molitorisova et al., 2021; Rajendran et al., 2014). Furthermore, kaempferol relaxes smooth muscles, dilates the pulmonary artery, and potentially improves respiratory function (Kianmehr & Khazdair, 2020; Mahobiya et al., 2018), thereby reducing the respiratory muscle load and oxygen cost during exercise.

One limitation of this study was that we demonstrated the significant effects of only a single intake of kaempferol. However, from a practical standpoint, it is necessary to compare the effects of a single intake with those of long‐term intake. Additionally, the effects of kaempferol intake on long‐term training adaptation should be investigated. To demonstrate its usefulness as a sports supplement, the effects of kaempferol must be verified during various types of exercises. Moreover, this study did not clarify the mechanism of kaempferol action, which remains unclear. To elucidate this mechanism, further experiments and human trials must be performed to clarify the involvement of NO and NOS in the presumed mechanism and identify any effects on mechanical efficiency. Furthermore, in addition to examining the effects of kaempferol on skeletal muscle, its effects on blood flow, cardiac function, and respiratory dynamics, which are presumed to be related to oxygen transport efficiency, must also be determined.

On the other hand, the strength of this study is that it is the first to observe the effects of kaempferol on oxygen demand during constant‐load exercises in well‐trained male athletes by a rigorous double‐blind trial. Additionally, consistent with previous studies, it can be unequivocally stated that kaempferol intake did not lead to negative physical symptoms (Akiyama et al., 2023; Ikeda et al., 2024; Ikeda, Gotoh‐Katoh, Yamamoto, et al., 2024; Okita et al., 2024).

## 5 CONCLUSIONS

This study provides the first evidence that kaempferol reduces oxygen demand and cost during submaximal constant‐load exercises and enhances exercise duration at maximal intensity without facilitating anaerobic metabolism in well‐trained athletes. Kaempferol intake may improve athletic ability by reducing the cardiopulmonary load during sports activities. However, the underlying mechanisms remain unclear. Therefore, further research is required to elucidate these mechanisms and investigate the multidisciplinary utility of kaempferol.

## AUTHOR CONTRIBUTIONS

K.O., T.M., and Y.I. conceived and designed the research; T.M., O.Y., and Y.I. performed the experiments; T.M. and Y.I. analyzed the data; K.O., T.M., and Y.I. interpreted the results of the experiments; K.O., T.M., and Y.I. prepared the figures; K.O. drafted the manuscript; Y.I. edited the manuscript. All authors approved the final version of the manuscript.

## CONFLICT OF INTEREST STATEMENT

The authors declare that there are no conflicts of interest, and the study results are presented clearly, honestly, and without fabrication, falsification, or inappropriate data manipulation.

## Supporting information


Table S1.

## Data Availability

The data supporting the findings of this study are available from the corresponding author upon request.
