# Correction to “Acute kaempferol ingestion lowers oxygen uptake during submaximal exercise and improves high‐intensity exercise capacity in well‐trained male athletes”

**DOI:** 10.14814/phy2.70518

**Published:** 2025-08-22

Okita, K., Mizokami, T., Yasuda, O., & Ikeda, Y. (2025, May). Acute kaempferol ingestion lowers oxygen uptake during submaximal exercise and improves high‐intensity exercise capacity in well‐trained male athletes. *Physiological Reports*. *13*(9), e70369.

In the final steps, we made errors in converting some figures and tables into journal format. There were errors in the respiratory rate graphs presented in Figure 4 and in the VAS data shown in Table 2.

**FIGURE 4:**
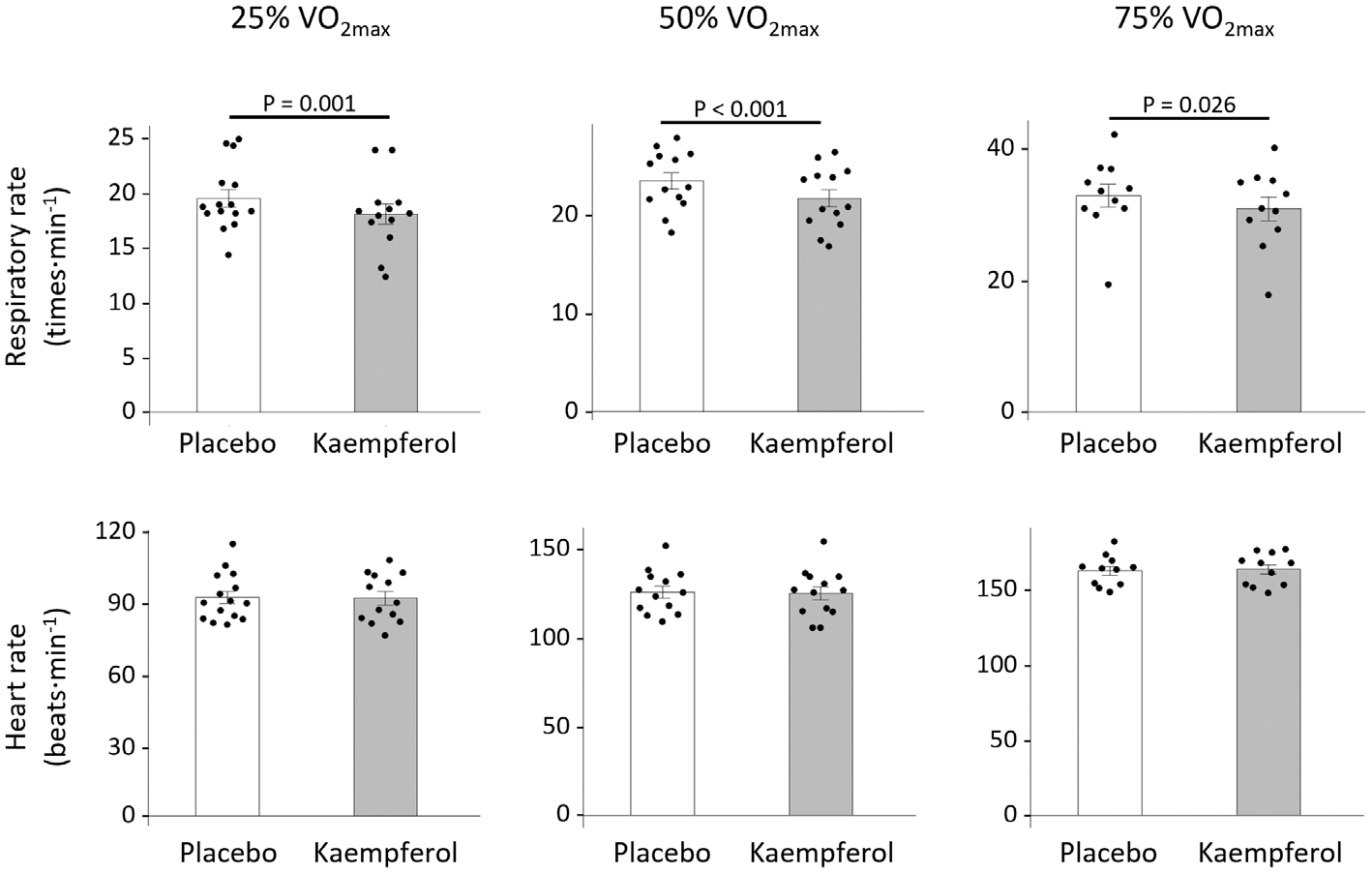
25% VO_2max_ condition: The *p*‐value was incorrectly reported as *p* < 0.001 and has been corrected to *p* = 0.001. 50% VO_2max_ and 75% VO_2max_ conditions: The graphs of respiratory rate mistakenly displayed VO_2_ data from Figure 3 instead of the correct respiratory rate data. The correct graphs are shown here.

**TABLE 2:** Regarding the VAS data at 75% VO_2max_, muscle fatigue and shortness of breath were incorrectly reported. The correct values are shown here. These changes do not affect the *p*‐values reported in the text.

Variables	Conditions	Numerical values		
		25% VO_2max_	50% VO_2max_	75% VO_2max_
VAS (cm)				
Muscle fatigue	Placebo	1.1 ± 0.2	3.9 ± 0.6	6.4 ± 0.8
	Kaempferol	0.9 ± 0.3	3.4 ± 0.7	6.6 ± 0.6
Shortness of breath	Placebo	1.3 ± 0.4	3.0 ± 0.3	5.8 ± 0.7
	Kaempferol	0.5 ± 0.1	1.7 ± 0.4	4.7 ± 0.7
RPE				
Tightness of the entire exercise	Placebo	7.8 ± 0.5	11.7 ± 0.6	15.1 ± 0.6
	Kaempferol	8.0 ± 0.5	11.6 ± 0.6	15.4 ± 0.5

We apologize for these errors which do not affect the scientific conclusions of the study.

