# Supplementary material for: Acute kaempferol ingestion lowers oxygen uptake during submaximal exercise and improves high‐intensity exercise capacity in well‐trained male athletes
Source: Physiol Rep. 2025 May 9;13(9):e70369. doi: 10.14814/phy2.70369 (PMC12064338; doi:10.14814/phy2.70369)
Supplement: Supplementary file 1 — Table S1. [file PHY2-13-e70369-s001.docx]

**Supplementary Table 1.** Absolute values of blood lactate during exercises.

| Exercise intensity | Test food | Blood lactate（mmol·L^-1^） | | | |
| --- | --- | --- | --- | --- | --- |
|  |  | Sampling points | | | ΔChanges |
|  |  | Before ingestion | Pre-exercise | Post-exercise | Post- from pre-exercise |
| 25% VO_2max_ | Placebo | 0.85 土 0.09 | 0.62 土 0.08 | 0.95 土 0.10* | 0.33 土 0.09 |
|  | Kaempferol | 0.71土 0.08 | 0.71 土 0.05 | 1.00 土 0.09* | 0.28 土 0.10 |
| 50% VO_2max_ | Placebo | 1.20土0.37 | 0.79 土 0.19 | 2.17 土 0.26* | 1.38 土 0.35 |
|  | Kaempferol | 0.73土0.08 | 0.65土0.04 | 2.04 土 0.26* | 1.38 土 0.24 |
| 75% VO_2max_ | Placebo | 1.21土0.10 | 1.45 土 0.11 | 6.75土0.98* | 5.30 土 1.01 |
|  | Kaempferol | 1.67土0.23 | 1.25 土 0.12 | 5.98 土 0.75* | 4.73 土0.80 |

*Data are presented as the mean ± SE. *P < 0.05 vs. pre-exercise.
